# High Prevalence of Human Cytomegalovirus Proteins and Nucleic Acids in Primary Breast Cancer and Metastatic Sentinel Lymph Nodes

**DOI:** 10.1371/journal.pone.0056795

**Published:** 2013-02-22

**Authors:** Chato Taher, Jana de Boniface, Abdul-Aleem Mohammad, Piotr Religa, Johan Hartman, Koon-Chu Yaiw, Jan Frisell, Afsar Rahbar, Cecilia Söderberg-Naucler

**Affiliations:** 1 Department of Medicine, Karolinska University Hospital, Stockholm, Sweden; 2 Department of Breast and Endocrine Surgery, Karolinska University Hospital, Stockholm, Sweden; 3 Department of Oncology-Pathology; Karolinska University Hospital, Stockholm, Sweden; University of Malaya, Malaysia

## Abstract

**Background:**

Breast cancer is a leading cause of death among women worldwide. Increasing evidence implies that human cytomegalovirus (HCMV) infection is associated with several malignancies. We aimed to examine whether HCMV is present in breast cancer and sentinel lymph node (SLN) metastases.

**Materials and Methods:**

Formalin-fixed paraffin-embedded tissue specimens from breast cancer and paired sentinel lymph node (SLN) samples were obtained from patients with (n = 35) and without SLN metastasis (n = 38). HCMV immediate early (IE) and late (LA) proteins were detected using a sensitive immunohistochemistry (IHC) technique and HCMV DNA by real-time PCR.

**Results:**

HCMV IE and LA proteins were abundantly expressed in 100% of breast cancer specimens. In SLN specimens, 94% of samples with metastases (n = 34) were positive for HCMV IE and LA proteins, mostly confined to neoplastic cells while some inflammatory cells were HCMV positive in 60% of lymph nodes without metastases (n = 35). The presence of HCMV DNA was confirmed in 12/12 (100%) of breast cancer and 10/11 (91%) SLN specimens from the metastatic group, but was not detected in 5/5 HCMV-negative, SLN-negative specimens. There was no statistically significant association between HCMV infection grades and progesterone receptor, estrogen receptor alpha and Elston grade status.

**Conclusions:**

The role of HCMV in the pathogenesis of breast cancer is unclear. As HCMV proteins were mainly confined to neoplastic cells in primary breast cancer and SLN samples, our observations raise the question whether HCMV contributes to the tumorigenesis of breast cancer and its metastases.

## Introduction

Breast cancer is the most common malignancy and a leading cause of cancer death in women worldwide (reviewed in [1]). A number of risk factors have been identified, including age, sex, long exposure to estrogen and a family history of breast cancer. However, recognized risk factors may be absent in 50–80% of patients [2], which has created an increased interest to identify additional risk factors that contribute to the disease.

Recent investigations have linked breast cancer to viral infections, such as Epstein–Barr virus (EBV) [3], mouse mammary tumor virus (MMTV) [4], human papillomavirus (HPV) [5] and most recently HCMV [6]. HCMV and human herpesvirus-8 (HHV-8) positivity in breast cancer was associated with lower relapse-free time and overall survival [5].

HCMV, a member of the β-herpesvirus family, is a common human pathogen infecting 70–90% of the world’s population. It remains latent for lifetime in its host after primary infection and reactivates periodically. HCMV can be transmitted through all bodily fluids, with transmission through breast milk playing a major role in the acquisition of the virus in early childhood [7]. Cell-free virus can be detected in the breast milk of over 90% of lactating seropositive women and 30–40% of one-year-old children have seroconverted for HCMV [8].

HCMV proteins and nucleic acids have been detected in several malignancies, including breast, colon, and prostate cancers as well as glioblastoma, medulloblastoma, mucoepidermoid cancer of the salivary gland and rhabdomyosarcomas (reviewed in [9], [10], [11]). Until recently, HCMV was believed to encode about 180 proteins that exhibit multiple biological activities that interfere with physiological functions in infected cells [12]. A recent study suggests that this number may in fact exceed 750 proteins [13], revealing that HCMV may be far more complex than previously believed. Most of these proteins are not essential for virus replication and are instead implied in various pathologies [12].

The role of HCMV in cancer is unclear but the virus exhibits both oncogenic and oncomodulatory properties by expressing HCMV proteins that can interfere with cellular processes [12], [14], [15]. The IE proteins are regulatory proteins expressed very early during the virus life cycle and regulate expression of viral and cellular genes. IE1 and IE2 proteins perform multiple functions; they are mutagenic when expressed in cells and are suggested to induce cellular transformation by a “hit and run” mechanism [16]. Recently, we showed that IE1 interacts with SP1 binding sites in the hTERT promoter and induces high telomerase activity, which is important in tumor transformation [17]. IE1 is also a kinase that regulates the activity of E2F and phosphorylates the Rb pocket proteins p107 and p130 [18], [19]. IE2 can bind to E2F and stimulate genes involved in cell cycle progression at the G1/S phase [20], but it does not drive cells into division [21]. IE2 also interacts with the p53 tumor suppressor protein and the Rb protein, modulating their activities [22], [23]. Furthermore both IE1 and IE2 proteins can prevent apoptosis by affecting death receptor signalling pathways such as the TNF-mediated death receptor-signalling pathway [24].

Late HCMV proteins are expressed later in the virus life cycle and are mainly structural proteins forming the virus particle. The HCMV tegument and envelope proteins, composed mainly of PP65, PP71, PP150, gB, gH, and gL play important roles in viral entry, gene expression, immune evasion, assembly and egress (Reviewed in [25]). Furthermore, HCMV gene products can further act in an oncomodulatory manner, through the blockage of cellular differentiation, induction of chromosomal instability, DNA mutations, induction of cellular migration and angiogenesis (reviewed in [9]). Furthermore, it has been shown that HCMV is capable of transforming certain types of mammalian cells [26], [27] and a gene region of HCMV is also shown to transform fibroblasts. More recently, transgenic mice expressing the HCMV gene US28 only in the intestine were shown to develop adenomas and adenocarcinomas [28], [29], further suggesting an oncogenic ability of HCMV. HCMV US2.8 is a chemokine receptor homologue that induces COX-2 expression, VEGF production, STAT3 phosphorylation and IL-6 production, all of which are relevant in tumor biology.

Although HCMV is found in several primary tumors, it is not known whether this virus remains active in metastatic cells. The aim of this study was to examine the prevalence of HCMV in 73 breast cancer and paired sentinel lymph node (SLN) specimens using a sensitive IHC method and Taqman PCR, and to determine if there is any association between HCMV infection with prognostic factors.

## Materials and Methods

### Clinical Samples

Patients were diagnosed between 2001 and 2003 at the Karolinska University Hospital, Stockholm, Sweden. All patients were prospectively included in a study concerning the use of the sentinel node biopsy as single axillary staging procedure and provided informed written consent for their participation. Results of this study are published elsewhere [30]. For the use of archived tissue specimens from these patients, permission from both the local ethics committee and the biobank at Karolinska Institute was granted. Samples were analyzed anonymously after a coding procedure, rendering it impossible for anyone except clinicians in charge to connect CMV data to the individual patient. A renewed consent procedure, involving direct contact with each individual many years after cancer treatment, was regarded unethical and therefore not performed. The ethics committee at Karolinska Institute explicitly approved this procedure with ethical number (No2008/628–31).

A total of 142 formalin-fixed paraffin-embedded tissue samples were obtained from 73 breast cancer patients with (n = 35) and without SLN metastasis (n = 38). Of these, paired SLN specimens were available from 34 and 35 patients, respectively. The diagnosis of breast cancer was confirmed by histological assessment at the pathology department, Karolinska University Hospital. The age of breast cancer patients ranged from 29 to 79 years (median 57 years).

### Immunohistochemical Analyses

Four µm thick paraffin sections were cut, dewaxed in xylene and rehydrated in a series of decreasing concentrations of ethanol (Apoteket pharmaci, Stockholm, Sweden)**.** After equilibrium in TBST (0.9% Nacl, 0.1 M Tris, pH7.5, TritonX-100, 2–3 drops/L), sections were post-fixed in 4% neutral buffered formalin (Apoteket pharmaci & Laboratorier AB, Sweden) prior to antigen unmasking with pepsin (BioGenex, San Ramon, CA, USA) at 37°C for 3 min before equilibrium in citrate buffer (pH 6.0, BioGenex). After that sections were incubated in 3% H_2_O_2_ (Merck, Germany) for 15 min to prevent endogenous peroxidase activity. Sections were then blocked for endogenous non-specific binding by the Avidin/Biotin blocking kit (Dako cytomation, Glostrup, Denmark), Fc-receptor blocker, for 20 min, followed by background buster for 20 min (both from the Innovex Sciences, Richmond, CA, USA). All incubations were performed at room temperature. Incubation with the primary antibody was done overnight at 4°C with anti-IE (1∶200; Chemicon Temecula, CA); anti-LA (1∶100; Chemicon, Temecula, CA); anti-cytokeratin (1∶100; Dakocytomation, Glostrup, Denmark) and TBST only (omitting primary antibody). The three-step horseradish peroxidase detection system was employed and the immunoreactivity revealed by chromogen diaminobenzidine (both from Innovex Biosciences) as described previously [31]. Negative controls included omitting primary antibody and isotype controls, the epithelial marker anti-cytokeratin served as a positive staining control.

To exclude cross reactivity with the HCMV IE and LA specific antibodies used in this study to other herpesviruses proteins, we collected EBV, herpes simplex virus type 1 and 2 (HSV-1, HSV-2) and HHV-8 infected cells, prepared cytospot slides and confirmed the presence of EBV, HSV-1, HSV-2 and HHV-8 proteins by immunocytochemistry using monoclonal mouse anti-EBV (EBNA2), polyclonal rabbit anti-HSV-1, HSV-2 (Dako cytomation, Glostrup, Denmark) and rat monoclonal antibody to HHV-8 (ORF73 LNA­1) (Advanced Biotechnoloies, Columbia, USA). We then used these cell samples and tested the reactivity of the HCMV-specific antibodies used in this study. We did not observe any immunoreactivity to EBV, HSV1, HSV2 and HHV-8 infected cells.

The staining results for HCMV were evaluated by estimating the percentage of IE and LA positive cells. Sections were graded as negative (0), grade I (<25% positive cells), grade II (25–49%), grade III (50–75%) or grade IV (>75%). Two investigators independently graded IHC positivity and a pathologist confirmed the results.

### TaqMan PCR

To confirm that HCMV nucleic acids were present in the pathological sections, DNA was extracted from 28 HCMV-positive or -negative tissue samples using the Picopure DNA extraction kit (Applied Biosystems, Branchburg, NJ) according to the manufacturer’s instructions. In brief, breast cancer or SLN tissue sections on slide were scraped with a sterilized blade after being dewaxed in xyelene, washed with 99% ethanol and air-dried. Then samples were digested with proteinase K in DNA extraction buffer for 24 hours at 65°C. Samples were heated to 95°C to deactivate the proteinase K before being subjected to TaqMan PCR. The TaqMan PCR was performed with 7900HT fast real-time PCR system from Applied Biosystems using primers and probes for the IE-gene (GTGACCCATGTGCTTATGACTCTAT, CTCAACATAGTCTGCAGGAACGT, FAM-TTGGTCACGGGTGTCTC-MGBNFQ) with RNase P as endogenous control (Applied Biosystems, Branchburg, NJ).

DNA samples from the SLN-positive (n = 8) and the SLN-negative (n = 8) groups (including breast (n = 4) and SLN (n = 4) samples each) were tested for other herpesviruses. All the DNA samples were used for detection of HSV-1, HSV-2 and HHV-8. Fifteen of these samples were also examined for EBV and 5 samples for varicella zoster virus (VZV) (due to lack of sufficient material for all tests). The virology department for clinical diagnostics at Karolinska University Hospital and the Swedish institute for communicable disease control (SMI) performed all these tests.

## Results

### High Prevalence of HCMV Antigens in Breast Cancer and Metastatic Sentinel Lymph Node Specimens

**Figure 1:**
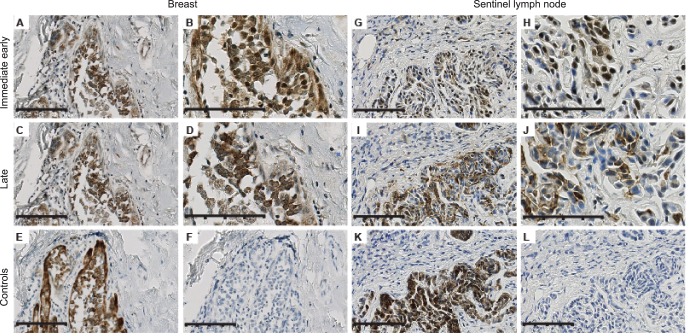
Immunohistochemistry of breast cancer and SLN samples. HCMV proteins were detected in the tissue sections from breast cancer patients (A–D) and tissue sections from sentinel lymph node (G–J) by immunohistochemistry. HCMV IE expression is confined to tumor cells both in breast and SLN specimens (A and G 20x; B and H 40x). (C, D, I, and J), same sections show immunoreactivity to HCMV LA protein (C, I 20x and D, J 40X). Cytokeratin was used as an epithelial marker and positive control (E, K) and omitting primary antibody was used as a negative control (F and L, 20x). Scale bars: (B, D, H, J, 100 **µ**m), (Others 80 **µ**m).

**Figure 2:**
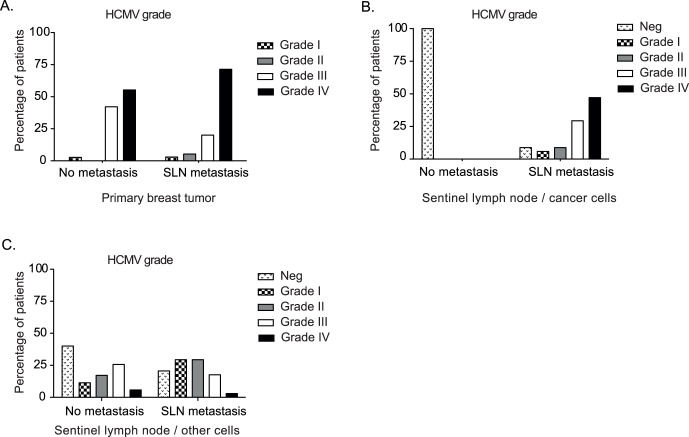
HCMV IE grade in cancer cells from breast and SLN, and in inflammatory cells from SLN samples. Patients were divided into 2 groups based on the presence or absence of SLN metastasis in both breast (A) and SLN (B and C) specimens. The positivity of HCMV was graded into four grades based on the percentage of HCMV IE positive cells in tumors from breast and paired SLN: Grade I (<25% cells positive for HCMV IE), grade II (25–49%), grade III (50–75%) and grade IV (>75%). The presence of HCMV IE-infected inflammatory cells in SLN specimen is shown in (C).

To determine whether HCMV was present in breast cancer tissue specimens and their paired sentinel lymph nodes, we examined archived tissue samples with an immunohistochemistry (IHC) assay using monoclonal antibodies against HCMV IE and LA proteins. Breast cancer tissue samples with paired specimens from SLN were obtained both from patients with SLN metastasis (n = 35, referred to as SLN-positive group) and without metastasis (n = 38, the SLN-negative group). HCMV IE and LA antigens were detected in all examined breast cancer specimens (n = 73) (Figure 1 and 2A). Both HCMV IE and LA proteins were detected in 32/34 (94%) SLN positive samples and in 20/35 (60%) in the SLN-negative group (P = 0.001). HCMV protein expression was mainly confined to metastatic tumor cells (Figure 1 and 2B), although HCMV-positive inflammatory cells were found in 79% of SLN samples with metastasis, few HCMV-positive inflammatory cells were also detected in 60% of metastasis-free SLN (Figure 2C and Table 1). One of HCMV negative patient samples in the SLN-positive group did not show cytokeratin-positive cells, suggesting that there were no tumor cells in this sample. We observed that more patients had higher HCMV infection grade in their primary tumor than in their SLN metastases (p = 0.0121) (Figure 2A and 2B).

**Table 1:** Immunohistochemical analysis of breast cancer and SLN samples.

HCMV Grade		
	SLN-Negative* n (%)	SLN-Positiven (%)
**HCMV IE in Breast cancer**NegativeGrade IGrade IIGrade IIIGrade IV	**n = 38**0 (0)1 (2.6)0 (0)16 (42.1)21 (55.3)	**n = 35**0 (0)1 (2.9)2 (5.7)7 (20)25 (71.4)
**HCMV LA in Breast cancer**NegativeGrade IGrade IIGrade IIIGrade IV	**n = 38**0 (0)8 (21.1)10 (26.3)14 (36.8)6 (15.8)	**n = 35**0 (0)5 (14.3)13 (37.1)12 (34.3)5 (14.3)
**HCMV IE in SLN (Cancer cells)**NegativeGrade IGrade IIGrade IIIGrade IV	**n = 35**35 (100)0 (0)0 (0)0 (0)0 (0)	**n = 34**3 (8.8)2 (5.9)2 (8.8)10 (29.4)16 (47.1)
**HCMV IE in SLN (Other cells)**NegativeGrade IGrade IIGrade IIIGrade IV	**n = 35**14 (40)4 (11.4)6 (17.1)9 (25.7)2 (5.7)	**n = 34**7 (20.6)10 (29.4)10 (29.4)6 (17.6)1 (2.9)
**HCMV LA in SLN (Cancer cells)**NegativeGrade IGrade IIGrade IIIGrade IV	**n = 35**35 (100)0 (0)0 (0)0 (0)0 (0)	**n = 34**3 (8.8)6 (17.6)5 (14.7)10 (29.4)10 (29.4)
**HCMV LA in SLN (Other cells)**NegativeGrade IGrade IIGrade IIIGrade IV	**n = 35**14 (40)9 (25.7)6 (17.1)6 (17.1)0 (0)	**n = 34**17 (50)10 (29.4)4 (11.8)3 (8.8)0 (0)

The positivity of HCMV was graded into four grades based on the estimated percentage of HCMV IE positive cells in tumors from breast and paired SLN: Grade I (<25% cells positive for HCMV IE), grade II (25–49%), grade III (50–75%) and grade IV (>75%), *n = number.

To exclude cross reactivity between HCMV IE and LA antibodies against other herpesviruses proteins, we collected EBV, HSV-1, HSV-2, and HHV-8 infected cells. We did not observe any reactivity to these cells with the HCMV-specific antibodies used in this study (data not shown). We also performed DNA analyses for other herpesviruses on four breast and four SLN samples in both groups (n = 16); we did not detect DNA of HSV-1, HSV-2, HHV-8, EBV (n = 15) or VZV (n = 5) in any of the samples, except for one obtained from the SLN-negative group that was EBV positive (data not shown).

To confirm the presence of HCMV in breast cancer and SLN samples, we performed TaqMan PCR using primers for the HCMV IE gene. DNA was extracted from paraffin sections of 12 tumors and 16 lymph nodes from the metastatic and non-metastatic groups. We detected HCMV DNA in 12/12 (100%) breast cancer specimens, and in 10/11 (91%) metastatic SLN specimens. We did not detect HCMV DNA in any of 5 examined SLN samples that were HCMV negative by IHC (data not shown).

### The HCMV Infection Level was not Correlated with Clinical Prognostic Factors

To examine whether the grade of HCMV infection was associated with known relevant clinical factors for breast cancer, such as SLN status, Estrogen Receptor alpha (ERα), Progesterone receptor (PR) and Elston grading (Her2-neu was only available for a small number of patients), we estimated the number of HCMV IE- and LA-positive cells using a four-grade scale. For HCMV IE, most breast cancer tissue specimens were categorized as grade III or IV, regardless of the categorization in the SLN-positive or -negative groups. We found that 32/35 (91%) and 37/38 (97%) of breast cancer specimens were grade III or IV in the SLN-positive and -negative groups, respectively (Table 1 and Figure 2A). Grade II and III were the most common grades for HCMV LA; 25/35 (71%) SLN-positive and 24/38 (63%) SLN-negative patient samples were LA positive (Table 1). In positive SLN, 26/34 (76%) samples were graded as HCMV IE grade III or IV; most metastatic cells were HCMV positive (Table 1, Figure 2B).

**Figure 3:**
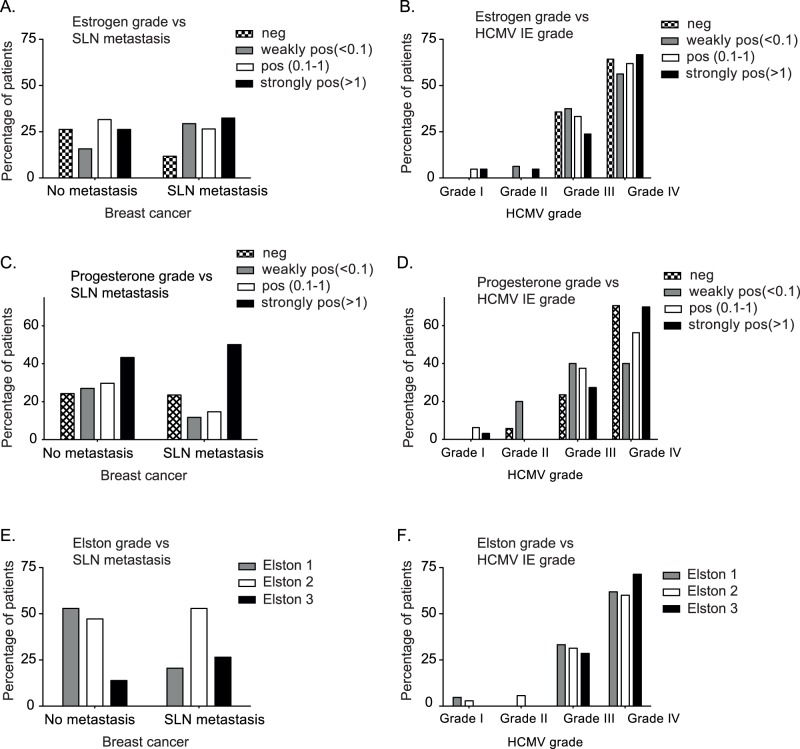
HCMV IE expression in breast cancer tissue and clinical prognostic markers. Expression of estrogen (A) and progesterone (B) receptor and Elston grading from breast cancer tissue of the SLN-negative and SLN-positive group are shown and are plotted in relation to HCMV IE grading (B, D and F) respectively.

ERα and PR negativity and high Elston grade are poor prognostic markers for breast cancer patients. Data for ERα, PR and Elston grading were obtained from clinical records (Table 2), but due to the low number of patients in each group only descriptive data with no further statistical analyses are reported (Figure 3 and Table 2).

**Table 2:** Patient characteristics.

Patients’ characteristics		
	SLN-Negativen (%)	SLN-Positiven (%)
**Age (Year)**	41–76	29–79
**Elston grade**123	14 (38.9)17 (47.2)5 (13.9)	7 (20.6)18 (52.9)9 (26.5)
**Estrogen grade**NegativeWeakly positive (<0.1)Positive (0.1–1)Strongly positive (>1)	10 (26.3)6 (15.8)12 (31.6)10 (26.3)	4 (11.8)10 (29.4)9 (26.5)11 (32.4)
**Progesterone grade**NegativeWeakly positive (<0.1)Positive (0.1–1)Strongly positive (>1)	9 (24.3)1 (2.7)11 (29.7)16 (43.2)	8 (23.5)4 (11.8)5 (14.7)17 (50)
**Proliferation grade**Low less than 10%High more than 10%	29 (85.3)5 (14.7)	24 (70.6)10 (29.4)
**Her2nu**NegativePositive	3 (75)1 (25)	14 (93.3)1 (6.7)
**Tumor type**DuctalLobularMixedOther	32 (88.9)4 (11.1)0 (0)0 (0)	22 (66.7)8 (24.2)2 (6.1)1 (3)
**Recurrence**Local recurrenceDistal recurrenceAxillary recurrence	4 (10.5)3 (7.9)2 (5.3)1 (2.6)	5 (14.3)0 (0)5 (14.3)0 (0)
**Death of breast cancer**	2 (5.3)	5 (14.3)
**Death**	3 (7.9)	6 (17.1)

**Figure 4:**
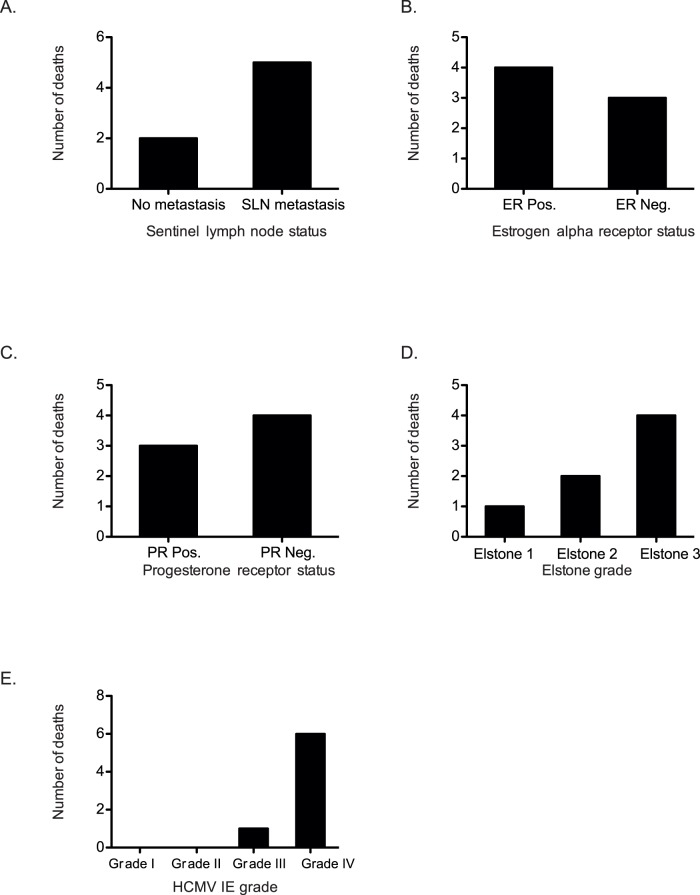
Death of breast cancer patients in relation to known prognostic markers and HCMV IE grade. Among 73 patients included in this study, 7 deaths were recorded in the SLN-negative (n = 2) and SLN-positive (n = 5) groups (A), 3 of 7 patients were negative for estrogen receptor (B), Progesterone expression were negative in 4 of 7 patients (C). 4 of 7 had high Elston grade (D), HCMV IE grade IV was observed in 6 of 7 patients (E).

Only 7 of 73 (9.5%) patients included in the study died of breast cancer, which did not allow for further statistical analyses of a possible association between deaths of breast cancer with HCMV infection grade. We noted that six of the patients who died of breast cancer had HCMV IE grade IV (n = 2 without metastasis, n = 5 with metastasis) and one had HCMV IE grade III (Figure 4E). The four cases with low HCMV IE expression (grades I and II, n = 4) were found among the survivors (Figure 4E).

## Discussion

Emerging evidence demonstrates a very high prevalence of HCMV proteins and nucleic acids in several malignancies originating in different organs. Here, we confirmed the recent observations by Harkins et al [32] demonstrating high HCMV protein expression in breast cancer. We found that 100% of primary breast cancer samples were HCMV positive, and for the first time report that virus protein expression could be detected in most neoplastic cells in sentinel lymph node metastases of breast cancer. These observations suggest that HCMV protein expression is maintained in most metastatic cells and so the role of the virus should be further evaluated to understand possible mechanisms contributing to breast cancer tumorigenesis and metastatic disease.

Infections have been proposed to be responsible for over 20% of all malignancies worldwide [33]. Recent investigations have linked viral infections such as EBV, MMTV [4], HPV [34] and HCMV [6] to breast cancer. It has been hypothesized that late exposure (in adulthood) to a common virus, such as HCMV, might be a potential risk factor for breast cancer [35]. Indeed, elevation of serum HCMV IgG levels was shown to precede the development of breast cancer [36]. Furthermore, Tsai et al showed that HCMV and HHV-8 were related to lower relapse-free time and overall poor survival of breast cancer [5], which further implies a link between HCMV and this tumor form.

The fact that HCMV is shed in breast milk, as well as in saliva, urine, cervical secretions and semen, implies that HCMV persistently infects epithelial cells [37]. Over 90% of lactating women secrete HCMV in breast milk, and 67% of normal glandular breast epithelium in patients who underwent mammoplasty were HCMV positive [6]. These observations suggest that HCMV is present in normal breast tissue. We found, however, that virus positivity is mainly restricted to tumor cells in established tumors and metastases, although it can be detected in some inflammatory cells. We detected HCMV proteins in all examined breast cancer samples, including both non-metastatic and metastatic tumors. Importantly, 94% of lymph node metastases of breast cancer were HCMV positive. The majority of breast cancer patients with sentinel node metastases had a primary tumor with HCMV IE grade IV. This was, however, also common in breast cancer specimens without SLN metastases.

Many studies imply that inflammation is associated with cancer [38], [39]. The inflammatory mediator COX-2 and its metabolites prostaglandins have been implicated as growth factors for tumor cells [40]. Many tumors, including breast cancer, are COX-2-positive, and high COX-2 expression levels are associated with poor clinical outcome [41]. Both selective and non-selective COX inhibitors decrease cancer incidence and show promise as anti-cancer agents (reviewed in [42]). Interestingly, HCMV induces inflammation and at the same time utilizes sophisticated strategies to escape immune recognition [43], [44]. The HCMV protein US28 is a constitutively active chemokine receptor homologue that induces COX-2 expression and results in STAT3 phosphorylation, which increases the production of VEGF and IL-6 and consequently induce tumor formation in vivo [45]. Recently, we showed that HCMV proteins were expressed in medulloblastoma and that virus infection induced COX-2 expression. Both aspirin and selective COX-2 inhibitors can efficiently prevent HCMV replication [46]. We showed that both antiviral drug valganciclovir and the specific COX-2 inhibitor celecoxib prevented HCMV replication in vitro, inhibited PGE2 production and significantly reduced medulloblastoma tumor cell growth both in vitro and in vivo [47]. In patients with glioblastoma, we found that low grade of HCMV infection in the tumor at diagnosis was strongly associated with prolonged survival [48]. These observations imply that HCMV may be involved in tumor development and that HCMV-targeted therapies may provide additional treatment options for patients with HCMV-positive tumors [43].

SLN status is currently the most powerful predictor of survival in breast cancer patients, largely influencing adjuvant treatment decisions [49], [50]. Loss of estrogen receptor alpha (ERα) and progesterone receptor (PR) expression are also predictors of poor survival [51]. Furthermore, the Elston-Ellis histopathological grading, assessing tubular formation, mitotic activity and nuclear pleomorphism, provides important prognostic information; Elston grade I tumors have a significantly better survival than those with grade II and III tumors [52]. We found that HCMV proteins were expressed in most SLN and that expression was mainly confined to tumor cells. HCMV could not be related to clinical prognostic factors or survival in this study, due to the low number of patients in each group. Although the finding of an infectious agent in tumor tissues does not provide evidence for its causal involvement in carcinogenesis, the presence of HCMV both in the primary breast tumor and in most SLN metastases, while adjacent healthy tissues remained HCMV negative, supports the hypothesis that HCMV may play an active role in tumorigenesis and metastasis of breast cancer.
